# Socio-demographic and socio-economic disparities in medical rehabilitation in Germany in 2019

**DOI:** 10.1093/eurpub/ckac131.036

**Published:** 2022-10-25

**Authors:** S Kniffert, O Stamm, A Heimann- Steinert

**Affiliations:** 1 BIH QUEST for Responsible Research, Berlin Institute of Health at Charité, Berlin, Germany; 2 Geriatrics Research Group, Charité – Universitätsmedizin Berlin, Berlin, Germany

## Abstract

Medical rehabilitation is a valuable component to restore physical and mental health, to prevent social isolation and to ensure a return to work. Due to the demographic change and the still increasing number of Post-Covid-19 patients, the demand for rehabilitation is still increasing. Latest, the COVID-19 pandemic elucidated that especially people from lower socio-economic backgrounds are disproportionally affected by health crisis. The aim of this study was to determine the influence of socio-demographic and socio-economic factors on quantitative exercise therapy in medical rehabilitation and to clarify the divergence of existing research findings. In our study we used data from 824.606 rehabilitation cases (German Federal Pension Insurance) and investigated the role of age and gender, marital status, social status and location towards inequalities in access to medical rehabilitation. Multiple linear regression and effect size calculation were used to show associations and to discuss the relation to clinical relevance. We were able to show a highly significant difference (P < 0.001) in access to exercise therapy in our study group. Patients aged 65+, women, single or widowed people, rehabilitants of low socioeconomic status or people located in the new federal states in Germany received shorter and less frequent exercise therapy. There are differences of up to 3.5 hours of treatment duration and 4.25 treatments per week, when disadvantaged social factors accumulate. However, despite the presented differences, the received treatments for the disadvantaged groups are still in the range of suggested minimum therapeutic requirements by the German Federal Pension Insurance. We could show a significant difference in rehabilitative therapy, which is solely based on social factors. There is an urgent need to draw special attention to the here discussed inequalities in access to medical rehabilitation for socially disadvantaged population and the overarching impact on society.

**Key messages:**

• The social inequality in medical rehabilitation to the detriment of the socially disadvantaged population that we have identified must give rise to clear changes in order to establish social justice.

• Equal opportunities and health quality assurance that address the individual needs of each patient should be the focus of socio-medical, policy development.

